# Lipoprotein cholesterol fractions are related to markers of inflammation in children and adolescents with juvenile idiopathic arthritis: a cross sectional study

**DOI:** 10.1186/s12969-016-0120-6

**Published:** 2016-11-11

**Authors:** Anna-Helene Bohr, Freddy Karup Pedersen, Claus Henrik Nielsen, Klaus Gottlob Müller

**Affiliations:** 1 Department of Paediatrics and Adolescent Medicine, Naestved Hospital, Rigshospitalet, Afs. 7821, Copenhagen N, Denmark; 2 Department of Paediatrics and Adolescent Medicine, Copenhagen University Hospital, Rigshospitalet, Denmark; 3 Institute for Inflammation Research. Center for Rheumatology and Spine Diseases, Copenhagen University Hospital, Rigshospitalet, Denmark; 4 JMC Research Unit, Copenhagen University Hospital, Rigshospitalet, Copenhagen, Denmark

**Keywords:** Juvenile chronic arthritis, JIA, Cholesterol-fractions, HDL-cholesterol, LDL- cholesterol, MRP8/14, BMI, Waist-to-height ratio, Premature atherosclerosis

## Abstract

**Background:**

The purpose of the study is to determine levels of total cholesterol (TC), low-density, and high-density lipoprotein fractions of cholesterol (LDLc and HDLc), in patients with juvenile idiopathic arthritis (JIA), and relate those to disease activity, overweight, and physical activity (PA), testing the hypothesis that the levels of cholesterol fractions are associated with inflammation as well as with overweight and low PA.

**Methods:**

Two hundred ten patients with JIA were included in this descriptive cross-sectional study. TC, LDLc, HDLc were measured, and associations with clinical disease activity (JADAS27), biomarkers of inflammation (myelo-related protein complex 8/14 (MRP8/14), C-reactive protein (CRP), and erythrocyte sedimentation rate (ESR)), body mass index (BMI), waist-to-height ratio (WtH ratio), and PA were explored.

**Results:**

Mean values for TC, LDLc, and HDLc in the patients were within the normal range for Danish Children. HDLc was negatively correlated with MRP8/14 (*r* = −0.343, CI −0.474 to −0.201, *p* < 0.0005) but was not related to overweight or PA. Neither TC nor LDLc showed any association with inflammation, overweight, or PA. MRP8/14 correlated positively with CRP, JADAS27 and WtH ratio (*r* = 0.277, CI 0.142 to 0.413, *p* = 0.001).

**Conclusions:**

Levels of cholesterol fractions in patients with JIA were found within the normal range. Nonetheless, the level of HDLc was negatively associated with the level of the inflammatory marker MRP8/14, which is in accordance with the concept of inflammation as an important driver for premature development of atherosclerosis in JIA. WtH ratio (a measure of central fatness) was not associated to HDLc, but to MRP8/14, suggestive of central fatness as an additional driving factor for the chronic inflammation in JIA.

## Background

Juvenile idiopathic arthritis (JIA) is a longstanding inflammatory arthritis of unknown origin in a child below 16 years of age. In the Nordic European countries is found an incidence of 15 per 100,000 children per year [1]. JIA includes several clinical subtypes, probably of different pathogenesis. The outcome in terms of functional ability has changed greatly during the last two decades due to progress in medical anti-inflammatory treatment; only few patients nowadays become functionally impaired during childhood or as young adults. However, a cure for JIA has not been found, and less than half of the patients achieve full and permanent remission [2–4].

Premature development of atherosclerosis is a well described feature of several chronic inflammatory diseases, including rheumatoid arthritis (RA) [5–7]. The clinical similarity between RA and JIA has given rise to several studies of the vasculature of patients with JIA, leaving no doubt that also JIA is associated with early signs of subclinical atherosclerosis [8, 9]. Persisting systemic inflammation may be a primary contributor, but other known risk factors for development of atherosclerosis, such as dyslipidemia, obesity, and sedentary behavior may contribute as well, and should be addressed prophylactically along with anti-inflammatory treatment.

In the general population, a high level of total cholesterol (TC), with a high fraction of low-density lipoprotein cholesterol (LDLc) and a low fraction of high-density lipoprotein cholesterol (HDLc), is associated with development of atherosclerosis [10]. Observational studies show that patients with RA have a nearly 50 % increased risk of experiencing clinically significant cardiovascular events compared to the general population [6, 7], but have a cholesterol profile different from the atherogenic profile described above. It is generally accepted that untreated RA, with the highest risk of clinically important atherosclerosis, is associated with low levels of HDLc and LDLc [10–12]. This is intriguing, as lipid deposits in the arterial intima are the hallmark of atherosclerosis.

Most previous studies of cholesterol fractions in JIA reported decreased levels of HDLc, while the level of LDLc varies between studies; a consistent pattern in regard to concomitant clinical or biochemical markers of inflammation is not evident in the literature [13–19].

Overweight and obesity in children and adolescents is typically associated with increased TC and decreased HDLc [20, 21]. The worldwide rise in the incidence of overweight and obesity reported in children and adolescents could also occur among patients with JIA and may also contribute to changes in cholesterol fractions seen in children with JIA [19].

A low level of physical activity (PA) may *per se* be associated with a raised level of TC [22]. A low level of PA in patients with JIA, as found in a recent study of PA in children with JIA [23], may possibly contribute to the divergent findings regarding lipid fractions in JIA.

The purpose of this study is to examine the levels in the blood of the cholesterol fractions (TC, LDLc, and HDLc), in children and adolescents with JIA, testing the hypothesis that the cholesterol fractions are correlated with inflammation as well as with overweight and PA.

## Methods

### Design

This is a descriptive cross-sectional study of blood cholesterol fractions, disease characteristics, anthropometrics, and objectively measured PA in patients with JIA. The patient population and level of PA was recently described in detail [23].

### Patients

All patients with JIA, 7 to 20 years of age, followed during a period from May 2011 to May 2013 at a population based out-patient specialized center for pediatric rheumatology covering Copenhagen and the eastern part of Denmark, were invited to participate. No one had significant chronic conditions other than JIA, especially no known conditions connected with dyslipidemia, and no patients received corticosteroid at the time of investigation, or had had recent intra-articular injection of corticosteroids. The patients were asked about family history of cardiovascular disease.

The patients were diagnostically sub-grouped in accordance with ILAR criteria [24].

**Fig. 1:**
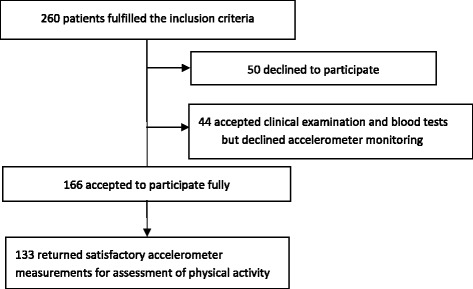
Included patients

Of 260 eligible patients, 50 declined to participate for logistical or social reasons not related to their disease. Forty-four patients declined accelerometer monitoring but accepted clinical evaluation and blood tests. Thus a total of 210 patients were available for clinical evaluation and analyses of inflammatory markers and cholesterol fractions in the blood; 166 accepted to participate in the whole investigation, including monitoring with accelerometer, which was done in the weeks after clinical assessment and blood tests. Of these, 133 patients returned accelerometer monitoring results of a sufficient quality for evaluation as defined below (Fig. 1).

### Clinical valuation of disease activity

All participants were assessed clinically by a pediatrician experienced in pediatric rheumatology and not involved in the investigation. The patients were seen as part of a routine control, no one was seen because of flare. Disease activity was described by a composite score, Juvenile Arthritis Disease Activity Score including evaluation of 27 joints (JADAS27) [25, 26]: *i)* number of joints with active inflammation: cervical spine, elbows, wrists, metacarpophalangeal joints 1–3, proximal interphalangeal joints 1–5, hips, knees, ankles, *ii)* global disease scoring by physician, *iii)* global disease scoring by patient or parent according to age, and *iv)* truncated erythrocyte sedimentation rate (tESR). JADAS27 is measured on a scale from 0–57.

Global disease activity was scored using a visual analog scale (VAS) from 0 (no sign of disease) to 10 (maximal burden of disease) by the physician and the patients/parents (according to age). Remission was assessed according to Wallace criteria [27].

### Markers of inflammation in blood

C-reactive protein (CRP) was measured (Roche/Hitachi cobas c-system, Mannheim, Germany) by turbidimetry after precipitation of agglutinated antibodies to CRP; the lower detection limit is 0.15 mg/l. Myelo-related-protein complex 8/14 (MRP8/14) was measured quantitatively in serum by use of a commercial ELISA kit (Bühlmann Laboratories AG, Schönenbuch, Switzerland) according to the manufacturer’s instructions. The principle is a sandwich technique with a monoclonal antibody specific to the MRP8/14 heterodimeric and polymeric complexes. Detection limit is < 0.4 mikrogram/ml. Analysis of MRP8/14 was done on serum of blood samples frozen immediately after separation and stored at −80°. ESR was used in the JADAS27 truncated (tESR) according to the formula given by Consolaro et al. [25]: after converting ESR values of ≤ 20 mm/h to 0 and ESR ≥ 120 to 120, tESR was calculated as (ESR (mm/h) - 20)/10 leaving a score of tESR from 0 to 10.

### Treatment

All the patients were either in treatment with methotrexate (MTX) and/or biological DMARDS: TNF-α-inhibitors, IL-6 receptor inhibitor (tocilizumab) or T-cell co-stimulation inhibitor (abatacept), or solely by regular intra-articular injections of corticosteroid.

### Anthropometry

Overweight and obesity was assessed by BMI and waist-to-height ratio (WtH ratio).

Weight was measured with light clothing to the nearest 0.1 kg and height without shoes to the nearest 0.1 cm. BMI was calculated as weight/squared height, and standard deviation score (BMI SDS) was calculated by comparison with Danish age and gender specific BMI values, transformed into normal distribution [28]. Waist circumference was measured with a non-stretchable tape, after expiration in a normal breathing rhythm, midway between the lower rib margin and the iliac crest. Pubertal stage (Tanner stage), was assessed by the patients or parents according to pictograms.

### Blood pressure

Blood pressure was measured with a Dinamap paediatric monitor (model XL, Critikon, Inc, Tampa, FL, USA) with the patient in a sitting position. The last two measurements out of three at each arm were used for calculation a mean systolic and a mean diastolic blood pressure. As blood pressure increases with age and is correlated to gender and height, the measured blood pressures (systolic or diastolic) were classified as normal (<90-percentile), pre-hypertensive (between 90 and 95 percentile) or hypertensive (>95 percentile) according to calculated percentiles in corresponding subgroups in data from a large group of normal American children of different age, sex and height, weight, and race [29].

### Lipids

TC, LDLc, and HDLc were measured in plasma using Roche modular by enzymatic colorimetry (Cobas c-system, Roche Diagnostics, Mannheim, Germany).

Blood lipids were measured in a non-fasting state, as in the Danish pediatric reference material [30].

### Accelerometry

Volume and pattern of PA was assessed during seven days by an accelerometer, ActiGraph model GT1M (ActiGraph Inc., Pensacola, FL, USA) a small, portable device worn fastened around the waist with an elastic band. The device measures vertical acceleration of the body and the reliability in measuring PA has been documented [31]. The device cannot be used during swimming or bathing. Reasons for not wearing the device and the time spent without it were noted, as was time spent on horseback riding and cycling, since these physical activities are not captured accurately by accelerometry [32].

The raw data were converted, after correction for non-wear time, by the ActiGraph software to *i)* mean counts per minute (cpm), a measure of average PA during total time of measurement, and *ii)* minutes per day spent in high PA (more than 3000 cpm), a cut-point routinely used by others, and *iii)* sedentary time assessed as average number of periods per day of more than 10 min with an activity of more than zero but less than 100 cpm. Only data collected during a minimum of 10 h per day for three days were considered reliable in assessing PA [22]. Periods of more than 10 min of zero count were considered as periods of “non-wear”.

### Primary endpoints

Disease activity (JADAS, CRP, MRP8/14), anthropometry (BMI, WtH ratio), and PA (cpm, sedentary time, high activity) correlated to TC, LDLc, and HDLc.

### Secondary endpoints

Correlations between cholesterol fractions and subgroup of JIA, treatment, disease duration, hypertension, gender, or Tanner stage were considered of explorative value.

### Statistical analyses

Summary statistics mean and standard deviation (SD), median and range, or numbers with percentages, were chosen as descriptive measures.

Associations were tested by T-test, Pearson bivariate correlation analyses, and analysis of variance (one-way ANOVA). As a significant correlation was found between MRP8/14 and WtH ratio and HDLc, we performed a multiple linear regression analysis. All variables were log_10_ transformed, because of a non-normal distribution. Spearman’s rho is calculated in analyses of JADAS, which could not be transformed to a normal distribution.

Significance (*p*) is given as two-tailed and with α = 0.004 after Bonferroni correction since we performed 12 independent tests on our primary endpoints: disease activity (JADAS/CRP), MRP8/14, anthropometry (BMI, WtH ratio), and PA (cpm, sedentary time, high activity) correlated to TC, LDLc, HDLc. Secondary endpoints were not corrected for multiple testing, as they were regarded as explorative.

All analyses were done in SPSS19, IBM Corp. IBM SPSS Statistics for Windows, Version 19.0. Armonk, NY: IBM Corp. USA, or in Excel 2010, Microsoft Corp. Redmond WA, USA.

## Results

### Patients, disease activity, and cholesterol fractions

The 210 included patients are described by gender, age, Tanner stage, anthropometrics, JIA subgroup, treatment, and disease duration (Table 1) and broadly similar to the features of the Nordic JIA cohort [3]. Mean age was 14.4 years, 58 % being in full puberty. Most patients were in treatment with biological DMARDs (68 %), by far most frequently with TNF-α inhibitors. Four patients (1.9 %) were in complete remission off medication, the treatment being withdrawn within the last 6 months. Seven of our patients had a family history of cardiovascular disease; the level of HDLc in these patients did not differ from the rest.

**Table 1:** Descriptive characteristics of the patients

Total number	210
Females/males	149 (71 %)/61 (29 %)
Age (years)	Mean 14.4 (SD 3.40) Median 15.00 (Range 7–21)
Height (m)	Mean 1.60 (SD 0.15) Median 1.62 (Range 1.14–1.87)
Weight (kg)	Mean 52.8 (SD 15.9) Median 53.8 (Range 18.5–97.0)
BMI SDS ^a^	Mean 0.4 (SD 1.17) Median 0.50 (Range −3.2–3.6)
Waist-to-height ratio (163 patients)	Mean 0.44 (SD 0.04) Median 0.43 (Range 0.35–0.62)
Tanner Stadium	121 (57.6 %) in Tanner Stage 5
JIA subgroup:	
- Systemic	6 (2.9 %)
- Persisting Oligoarticular	65 (31 %)
- Polyarticular, RF negative	53 (25 %)
- Polyarticular, RF positive	8 (3.8 %)
- Psoriasis associated	21 (10.0 %)
- Enthesitis associated	56 (26.7 %)
- Undifferentiated	1 (0.5 %)
- Anti-Nuclear Antibodies (ANA) positive	32 (15.2 %)
- HLA-B27 positive	40 (19 %)
Treatment	
- Methotrexate	40 (19 %)
- Biologics +/− Methotrexate:	142 (68 %)
either TNF-α inhibitors	130
or IL-6 receptor inhibitor	4
or T-cell co-stim inhibitor CTLA-4-Ig	8
- Solely by intra-articular injections	13 (6.2 %)
- None	10 (4.8 %)
- No information	4 (1.9 %)
Disease duration ^b^ in years	Mean 6.1 (SD 3,7) Median 5.2 (Range 0.5–17.3)
Complete remission off medication	4 (1.9 %)

Summary statistics: Number with percentage in brackets, mean with SD in brackets, and median with range in brackets

^a^ *BMI SDS* Body mass Index Standard Deviation Score, gender and age adjusted

^b^Disease duration = duration of symptoms

The group of patients that refused participation (no. 50) was comparable with the 210 participating patients with regard to age, gender and subgroup of JIA. The group that participated only partly and the group that participated fully were comparable also in the distribution of BMI and actual disease activity (data not shown).

Data on clinical disease activity and markers of inflammation in the blood are presented in Table 2. Numbers of inflamed joints, physician global VAS scores, ESR and CRP all indicate a general low level of disease activity in the patients.

**Table 2:** Markers of disease activity

Number of inflamed joints (0–27)	Mean 1.17 (SD 2.26) Median 0.0 (Range 0–24)
Physician global VAS (0–10)	Mean 1.13 (SD 1.28) Median 1.00 (Range 0–6.5)
Patient/parent global VAS (0–10)	Mean 1.67 (SD 2.05) Median 0.8 (Range 0–10)
ESR (mm/h)	Mean 10.4 (SD 7.8) Median 9.0 (Range 1–58)
Truncated ESR (tESR) (mm/h)	Mean 0.08 (SD 0.40) Median 0.00 (Range 0–3,8)
CRP (mg/l)	Mean 1.98 (SD 3.64) Median 0.61 (Range 0.16–31.0)
JADAS27 (0–57) ^a^	Mean 4.2 (SD 4.1) Median 3.2 (Range 0–21)
MRP8/14 (μg/ml)	Mean 1.34 (SD 1.22) Median 1.03 (Range 0.18–13.75)
Tendinitis and/or entesitis	47 (22.4 %)

Summary statistics: Number with percentage in brackets, mean with SD in brackets, and median with range in brackets

^a^JADAS27 (0–57) (169 patients): a composite score of: number of joints with active disease (max 27), Physician global VAS, patient/parent global VAS and truncated ESR (tESR)

Mean values for TC, LDLc, and HDLc were all within the normal range for Danish Children [30] (Table 3).

**Table 3:** Cholesterol fractions and triglyceride

		Danish pediatric reference intervals [30]
Total cholesterol (TC) mmol/l	Mean 3.94 (SD 0.72)	2.74–5.33
High Density Lipoprotein fraction of cholesterol (HDLc) mmol/l	Mean 1.48 (SD 0.35)	1.0–2.1
Low Density Lipoprotein fraction of cholesterol (LDHc) mmol/l	Mean 2.16 (SD 0.58)	1.1–3.6

Summary statistics: Number with percentage in brackets, mean with SD in brackets

### Correlation between primary and secondary outcomes and TC, HDLc, or LDLc

We found a significant negative correlation between HDLc and the inflammatory marker MRP8/14 with a Pearson correlation coefficient (r) of – 0.343, (*p* < 0.0005), while the correlation between HDLc and CRP and WtH ratio did not reach significance at the Bonferroni corrected level. HDLc and JADAS27 did not correlate significantly and we found no significant correlations between LDHc or TC and MRP8/14 or any other marker of inflammation. Neither TC nor cholesterol fractions correlated significantly with subgroups of JIA, treatment, duration of disease, gender, or Tanner stage. (Table 4).

**Table 4:** Associations between cholesterol fractions and disease characteristics, fatness, and physical activity *P* values with correlation coefficients, and 95 % confidence interval in brackets

Patients	TC mmol/l	HDLc mmol/l	LDLc mmol/l
Gender T-test	0.05	0.19	0.12
Tanner stage (≤4 or = 5) T-test	0.87	0.51	0.85
Subgroup of JIA ANOVA	0.05	0.85	0.09
Treatment ^a^ ANOVA	0.83	0.68	0.98
Disease duration Bivariate correlation	0.93	0.19	0.84
Primary endpoints:			
MRP 8/14 Bivariate correlation, *p* value Pearson correlation coefficient, r 95 % confidence interval	0.20	<0.0005* −0.343 (−0.474 to −0.201)	0.92
CRP Bivariate correlation, *p* value Pearson correlation coefficient, r 95 % confidence interval	0.83	0.043 −0.167 (−0.319 to–0.015)	0.63
JADAS 27 ^b^ Nonparametric correlation	0.66	0.72	0.92
Waist-to-height ratio Bivariate correlation, *p* value Pearson correlation coefficient r 95 % confidence interval	0.824	0.018 −0.187 (−0.345 to–0.033)	0.199
BMI SDS Bivariate correlation	0.15	0.13	0.49
Counts per minute (cpm) (133 patients) Bivariate correlation	0.23	0.25	0.33
Sedentary time (minutes/day) (133 patients) Bivariate correlation	0.23	0.61	0.11
High activity (minutes/day) (133 patients) Bivariate correlation	0.15	0.21	0.17

*TC* total cholesterol, *HDLc* high density fraction of cholesterol, *LDLc* low density fraction of cholesterol

*Significance (*p*) is given as two-tailed and α selected after Bonferoni correction as 0.004 (12 independent tests)

^a^Treatment: Methotrexate versus TNF-α inhibitor versus IL-6 receptor inhibitor versus T-cell co-stimulation inhibitor CTLA-4-Ig

^b^JADAS27 (0–57): a composite score of: number of joints with active disease (max 27), Physician global VAS, patient/parent global VAS and truncated ESR (tESR)

All parametric bivariate correlations have been performed after log10 transformation

### Correlations between inflammatory markers

MRP8/14 correlated positively and significantly with CRP (*r* = 0.480, *p* < 0.0005) and with JADAS27 (rho = 0.221, *p* = 0.006) (Table 5). To our knowledge no studies provide normal values of MRP8/14 in children.

**Table 5:** Correlation between the inflammatory marker MRP8/14 and CRP (log10 transformed) and JADAS27

MRP8/14 and CRP Pearson bivariate correlation	*r* = 0.480 (95 % CI 0.347–0.598)	*p* < 0.0005
MRP8/14 and JADAS27 Nonparametric correlation	rho = 0.221 (95 % CI 0.064–0.373)	*p* = 0.006

### Overweight and obesity and correlation to cholesterol and inflammation

Thirty percent of the patients had a BMI SDS above 1 SD, and 7.6 % had a BMI SDS above 2 SD compared to the Danish age and gender adjusted reference material [28]. Only 5 patients had a BMI SDS below 2 SD.

Prehypertension defined as a diastolic or systolic blood-pressure between the 90. and 95. percentile, was seen in 17 % of the patients with an unsurprising association to BMI but with no correlation to MRP8/14.

Neither BMI nor WtH ratio showed a significant correlation to TC, HDLc, or LDLc (Table 4). As a significant correlation was found between WtH ratio and MRP8/14 (Table 6) we performed a multiple linear regression analysis of HDLc as dependent on the independent variables WtH ratio and MRP8/14. WtH ratio did not add significantly to the prediction of HDLc (β = −0.069, *p* = 0.395) while MRP8/14 remained significant (β = −0.330, *p* < 0.0005) (Table 7).

**Table 6:** Pearson correlation between inflammatory markers (MRP8/14 and CRP) and markers of fatness (waist-to-height ratio and BMI SDS)

	MRP8/14	CRP
Waist-to-height ratio (WtH ratio)	*r* = 0.277 *p* = 0.001	*r* = 0.16 *p* = 0.051
BMI SDS	*r* = 0.128 *p* = 0.082	*r* = 0.128 *p* = 0.081

All variables have been log10 transformed

**Table 7:** Multiple linear regression analysis of HDLc as dependent of MRP8/14 and WtH ratio

Dependent	Independent	B	R	Standardized coefficient 95 % Confidence Interval	Level of Significance
HDLc	MRP8/14	- 0.330	- 0.349	- 0.169 to - 0.068	<4 × 10^−6^
HDLc	Waist-to-height ratio	- 0.069	- 0.160	- 0.553 to 0.220	0.395

B Standardized regression coefficient

R multiple correlation coefficient

All variables have been log10 transformed

We found no association between central fatness, measured by WtH ratio, and medication (data not shown).

### PA and correlations to cholesterol

Neither TC nor LDLc nor HDLc cholesterol fractions correlated with time spent on sedentary PA or with high PA (Table 4).

## Discussion

In our group of patients with JIA most had minimal disease activity, and TC, HDLc and LDLc were found within the normal range. Nevertheless, HDLc showed a negative correlation with MRP8/14, a sensitive marker of inflammation. In contrast, neither TC nor LDLc showed any significant correlation to markers of inflammation.

Our finding of a significant correlation between HDLc and a marker of inflammation is in agreement with two previous findings in JIA [14, 15]; both found a negative correlation between HDLc and the inflammatory marker CRP. Furthermore a study by Shen et al. [33] on 58 patients with JIA showed a significant increase in HDLc in parallel with a decrease in CRP in 31 patients with inactive disease after 18 months of anti-inflammatory treatment; the level of LDLc did not change. In contrast, another intervention study of patients with active JIA [34] showed a more traditional atherogenic lipid profile, with an increased total LDLc and normal HDLc at baseline. After 12 months of anti-inflammatory treatment, the investigators found a large decline in inflammatory markers concomitant with a relatively small decline in LDLc and no change in HDLc.

Most of the patients included in our study had low disease activity according to JADAS27 score, CRP, and ESR. In JIA with low to moderate disease activity, classical biomarkers of inflammation (CRP, ESR) are most often not increased. In order to capture a possible association between cholesterol fractions and low grade inflammation, we also measured MRP8/14. MRP 8 (the same as S100A8) and 14 (S100A9) are calcium-binding, potent pro-inflammatory proteins produced locally by activated phagocytic myeloid cells. MRP8/14 is released as a stable heterodimer into the circulation, where it acts as a pro-inflammatory mediator, promoting activation of inflammatory cells through binding to Toll-like receptors resulting in the production of cytokines such as interleukin-1β and IL-6 that, unimpeded, amplify and perpetuate the inflammation [35, 36]. The MRP8/14 dimer is measurable in the synovial fluid and the blood, and is correlated with measures of disease activity and risk of relapse after stopping anti-inflammatory medication in JIA [37–40]. While pro-inflammatory cytokines in JIA, such as TNF- α, are easily degraded and found at low concentration in the blood, MRP8/14 is a more abundant and more stable protein also at room temperature. In this study, MRP8/14 correlated significantly with JADAS27 and CRP.

The aforementioned association between inflammation and low levels of HDLc in active RA has been demonstrated in observational studies; no clear causality has been found. However, HDLc particles are important transporters of excess cholesterol from cells, e.g. macrophages in the arterial intima, to the liver (“reverse cholesterol transport”) [41]. Insufficient clearance of cholesterol could partly explain why atherosclerosis is seen in RA patients. Not only the level of HDLc, but also its function may be influenced by inflammation. Upon treatment of patients with RA with an anti-IL-6 R inhibitor, McInnes et al. [42] found a decrease in the binding of the acute phase reactant, serum amyloid A to HDL particles, as well as an increase in paraoxonase, an antioxidant enzyme connected to HDLc. Thus, HDL particles were reverted from a dysfunctional and pro-inflammatory state to a normal cholesterol-carrying state after anti-inflammatory treatment.

Overweight, defined as BMI SDS above 1, was seen in 30 % of our patients when compared to the Danish gender and age adjusted reference material [28]; neither BMI nor WtH ratio correlated with cholesterol fractions. However, we found a positive correlation between WtH ratio and MRP8/14. In both genders and regardless of age, WtH ratio is associated not only with BMI but also, independently of BMI, with other risk factors for development of cardiovascular disease, like hypertension and dyslipidemia, [43, 44]. WtH ratio reflects central, visceral fatness, which is considered the place of origin for development of the low-grade inflammation known to be associated with overweight and obesity. Central fatness may thus influence cardiovascular health in children with JIA by contributing to a continuing low-grade inflammation.

The lipids are measured in a non-fasting state. Older investigations are primarily built on lipids measured in a fasting state, but blood sampling after overnight fasting pose a practical problem in investigations of children living far from laboratories doing the blood sampling. Besides, children are most often in a non-fasting state, and thus the non-fasting levels of lipids may have the highest relevance as risk factors for endothelial damage and cardiovascular disease. In adults, TC and HDLc vary less than 2 % with fasting time; LDHc varies up to 10 % [45]. The Danish Pediatric reference intervals for lipids are based on blood samples taken from non-fasting children and adolescents.

Although the correlation between HDLc and MRP8/14 was highly significant in our study, the correlation coefficient was only moderate as in the study by Shen et al. [33], indicating influence by other factors than MRP8/14. In a review on PA and cardiovascular risk factors in children, Andersen et al. [46] suggested a positive effect of PA on HDLc, with no effect on LDLc and TC. Our study does not support this, as we found no significant correlations between PA and HDLc or LDLc. However, we did not measure fitness. Fitness is built up by regular PA and may thus be a better marker of general health and have a more significant influence on lipids in the blood, than the more sporadic PA that we were able to measure [47].

The present study has other important limitations. As some of the subgroups had a low number of patients, we have not performed any analysis concerning subgroups of JIA. Considering JIA as a uniform group, especially including sJIA, characterized by high levels of inflammatory mediators, may have inflated the found correlation between MRP8/14 and HDLc. Likewise we cannot exclude that hidden confounders correlated to the pathogenesis of specific subtypes, and not related to disease activity *per se*, may have influenced the findings. We did not find any correlation between treatment regimes and the cholesterol fractions, but we did not look at the specific doses of medicine, which could be of importance, as some medicine may have specific effect on the cholesterol fractions besides the anti-inflammatory effect. Most of our patients received effective anti-inflammatory treatment and had low disease activity. Looking at the level of HDLc before and during anti-inflammatory treatment would be important in future studies.

A cross-sectional descriptive study is at best able to demonstrate associations; causality must be tested in subsequent controlled longitudinal trials. In a setting of updated clinical and biological information, correlations can, nevertheless, have immediate clinical relevance. A strength of our study is that we have looked at a large community-based, representative, and well-described group of patients and have used a sensitive marker of inflammation (MRP8/14).

At present there is evidence that patients with JIA have an increased risk for early development of subclinical atherosclerosis [8, 9]. No long-term follow-up studies of adults with JIA have yet shown an increased risk of clinically important cardiovascular events in early adult life, underscoring that development of atherosclerosis is a complex process. Availability of effective anti-inflammatory medication and a strategy favoring an early and aggressive use of this may well have a beneficial effect also on vascular health, but adverse lifestyle factors should still be taken into consideration.

## Conclusions

Levels of cholesterol fractions in patients with JIA were found within the normal range. Nonetheless, the level of HDLc was negatively associated to the level of the inflammatory marker MRP8/14, which is in accordance with the concept of inflammation as an important driver for premature development of atherosclerosis in JIA. WtH ratio (a measure of central fatness) was not associated to HDLc, but to MRP8/14, suggestive of central fatness as an additional driving factor for the chronic inflammation in JIA.

## Key messages

Even in clinically well-controlled JIA, the level of HDLc is negatively associated to the level of the inflammatory marker MRP8/14.

Central fatness correlates independently to the level of MRP 8/14 and may thus exacerbate the chronic inflammation in JIA.

## Abbreviations

BMI: Body mass index
CRP: C-reactive protein
DMARD: Disease modifying anti-rheumatologic drugs
ESR: Erythrocyte sedimentation rate
HDLc: High-density lipoprotein fractions of cholesterol
JADAS27: Juvenile Arthritis Disease Activity Score including evaluation of 27 joints
JIA: Juvenile idiopathic arthritis
LDLc: Low-density lipoprotein fractions of cholesterol
MRP8/14: Myelo-related protein complex 8/14
PA: Physical activity
RA: Rheumatoid arthritis
TC: Total cholesterol
WtH ratio: Waist-to-height ratio

## References

[1] Berntson L, Andersson GB, Fasth A, Herlin T, Kristinsson J, Lahdenne P (2003). Incidence of juvenile idiopathic arthritis in the Nordic countries. A population based study with special reference to the validity of the ILAR and EULAR criteria. J Rheumatol.

[2] Minden K (2009). Adult outcomes of patients with juvenile idiopathic arthritis. Hormone Res.

[3] Nordal E, Zak M, Aalto K, Berntson L, Fasth A, Herlin T (2011). Ongoing disease activity and changing categories in a long-term nordic cohort study of juvenile idiopathic arthritis. Arthritis and rheumatism.

[4] Selvaag AM, Aulie HA, Lilleby V, Flatø B. Disease progression into adulthood and predictors of long-term active disease in juvenile idiopathic arthritis. Ann Rheum Dis. 2016;75(1):190–5. 10.1136/annrheumdis-2014-206034 25362042

[5] Kristensen SL, Ahlehoff O, Lindhardsen J, Erichsen R, Jensen GV, Torp-Pedersen C (2013). Disease activity in inflammatory bowel disease is associated with increased risk of myocardial infarction, stroke and cardiovascular death – a Danish nationwide cohort study. PLOS ONE.

[6] Avina-Zubieta JA, Thomas J, Sadatsafavi M, Lehman AJ, Lacaille D (2012). Risk of incident cardiovascular events in patients with rheumatoid arthritis: a meta-analysis of observational studies. Ann Rheum Dis.

[7] Lindhardsen J, Ahlehoff O, Gislason GH, Madsen OR, Olesen JB, Torp-Pedersen C (2011). The risk of myocardial infarction in rheumatoid arthritis and diabetes mellitus: a Danish nationwide cohort study. Ann Rheum Dis.

[8] Coulson EJ, Ng W, Goff I, Foster HE (2013). Cardiovascular risk in juvenile idiopathic arthritis. Reumatology.

[9] Bohr A, Fuhlbrigge RC, Pedersen FK, de Ferranti SD, Müller K (2016). Premature subclinical atherosclerosis in children and young adults with juvenile idiopathic arthritis. A review considering preventive measures. Pediatr Rheumatol.

[10] Robertson J, Peters MJ, McInnes IB, Sattar N (2013). Changes in lipid levels with inflammation and therapy in RA: a maturing paradigm. Nat Rev Rheumatol.

[11] Liao KP, Cai T, Gainer VS, Cagan A, Murphy SN, Liu C (2013). Lipid and lipoprotein levels and trend in rheumatoid arthritis compared to the general population. Arthritis Care Res.

[12] Myasoedova E, Crowson CS, Kremers HM, Roger VL, Fitz-Gibbon PD, Therneau TM, Gabriel SE (2011). Lipid paradox in rheumatoid arthritis: the impact of serum lipid measures and systemic inflammation on the risk of cardiovascular disease. Ann Rheum Dis.

[13] Ilowite NT, Samuel P, Beseler L, Jacobsen MS (1989). Dyslipoproteinemia in juvenile rheumatoid arthritis. J Pediatrics.

[14] Bakkaloglu A, Kirel B, Ozen S, Saatçi U, Topaloĝlu Beşbaş N (1996). Plasma lipids and lipoproteins in juvenile chronic arthritis. Clin Rheumatol.

[15] Tselepis AD, Elisaf M, Besis S, Karabina SP, Chapman MJ, Siamopoulou A (1999). Association of the inflammatory state in active juvenile rheumatoid arthritis with hypo-high density lipoproteinemia and reduced lipoprotein-associated platelet-activating factor acetylhydrolase activity. Arthritis Rheum.

[16] Urban M, Pietrewicz E, Górska A, Glowinska B (2004). lipids and homocysteine level in juvenile idiopathic arthritis (article in Polish with an english abstract). Pol merkur Lekarski.

[17] Conçalves M, D’Almeida V, Guerra-Shinohara EM, Galdieri LC, Len CA, Hilário MOE (2007). Homocysteine and lipid profile in children with juvenile idiopathic arthritis. Pediatr Rheumatol.

[18] Marangoni RG, Hayata AL, Borba EF, Azevedo PM, Bonfá E, Goldenstein-Schainberg C (2011). Decreased high-density lipoprotein cholesterol levels in polyarticular juvenile idiopathic arthritis. Clinics.

[19] Glowińska-Olszewska B, Bossowski A, Dobreńko E, Hryniewicz A, Konstantynowicz J, Milewski R (2013). Subclinical cardiovascular system changes in obese patients with juvenile idiopathic arthritis. Mediat Inflamm.

[20] Nielsen TRH, Gamborg M, Fonvig CE, Kloppenborg J, Hvidt KN, Ibsen H, Holm J (2012). Changes in lipidemia during chronic care treatment of childhood obesity. Childhood Obes.

[21] Skinner AC, Perrin EM, Moss LA, Skelton JA (2015). Cardiometabolic risks and severity of obesity in children and young adults. N Engl J Med.

[22] Andersen LB, Harro M, Sardinha LB, Froberg K, Ekelund U, Brage S (2006). Physical activity and clustered cardiovascular risk in children: a cross-sectional study (The European Youth Heart Study). Lancet.

[23] Bohr A, Nielsen S, Müller K, Pedersen FK, Andersen LB (2015). Reduced physical activity in children and adolescents with Juvenile Idiopathic Arthritis despite satisfactory control of inflammation. Pediatr Rheumatol.

[24] Petty RE, Southwood TR, Manners P, Baum J, Glass DN, Goldenberg J (2004). International league of associations for rheumatology classification of juvenile idiopathic arthritis. J Reumatol.

[25] Consolaro A, Ruperto N, Bazso A, Pistorio A, Magni-Manzoni S, Filocamo G (2009). for PRINTO. Development and validation of a composite disease activity score for juvenile idiopathic arthritis. Arthritis Care Res.

[26] Nordal EB, Zak M, Aalto K, Berntson L, Fasth A, Herlin T, Lahdenne P, Nielsen S, Peltoniemi S, Straume B, Rygg M (2012). Validity and predictive ability of the juvenile arthritis disease activity score based on CRP versus tESR in a Nordic population-based setting. Ann Rheum Dis.

[27] Wallace CA, Giannini EH, Huang B, Itert L, Ruperto N for CARRA and PRCSG and PRINTO (2011). American College of Rheumatology provisional criteria for defining clinical inactive disease in select categories of juvenile idiopathic arthritis. Arthritis Care Res.

[28] Tinggaard J, Aksglaede L, Sørensen K, Mouritsen A, Wohlfahrt-Veje C, Hagen CP (2014). The 2014 Danish references from birth to 20 years for height, weight and body mass index. Acta Paediatr.

[29] American Academy of Pediatrics (AAP) guidelines (2004). The fourth report on the diagnosis, evaluation, and treatment of high blood pressure in children and adolescents. Pediatrics.

[30] Hilsted L, Rustad P, Aksglæde L, Sørensen K, Juul A (2013). Recommended Nordic paediatric reference intervals for 21 common biochemical properties. Scand J Clin Lab Invest.

[31] Ekelund U, Sjöström M, Yngve A, Poortvliet E, Nilsson A, Froberg K (2001). Physical activity assessed by activity monitor and doubly labeled water in children. Med Sci Sports Exerc.

[32] Tarp J, Andersen LB, Østergaard L. Quantification of underestimation of physical activity during cycling to school when using accelerometry. J Phys Act Health. 2015;12(5):701–7. 10.1123/jpah.2013-0212 24906079

[33] Shen C, Yao T, Yeh K, Huang J (2013). Association of disease activity and anti-rheumatic treatment in juvenile idiopathic arthritis with serum lipid profiles: a prospective study. Semin Arthritis Rheum.

[34] De Sanctis S, Marcovecchio ML, Gaspari S, Del Torto M, Mohn A, Chiarelli F, Breda L (2013). Etanercept improves lipid profile and oxidative stress measures in patients with juvenile idiopathic arthritis. J Rheumatol.

[35] Kessel C, Holzinger D, Foell D (2013). Phagocyte-derived S100 proteins in autoinflammation: putative role in pathogenesis and usefulness as biomarkers. Clinical Immunology.

[36] Holzinger D, Kessel C, Omenetti A, Gattorno M (2015). From bench to bedside and back again: translational research in autoinflammation. Nat Rev Rheumatol.

[37] Foell D, Frosch M, Sorg C, Roth J (2004). Phagocyte-specific calcium-binding S100 proteins as clinical laboratory markers of inflammation. Clinica Chimica Acta.

[38] Holzinger D, Frosch M, Kastrup A, Prince FHM, Otten MH Van Suijlekom-Smit WA (2012). The toll-like receptor 4 agonist MRP8/14 protein complex is a sensitive indicator for disease activity and predicts relapses in systemic-onset juvenile idiopathic arthritis. Ann Rheum Dis.

[39] García-Arias M, Pascual-Salcedo D, Ramiro S, Ueberschlag M, Jermann TM, Cara C (2013). Calprotectin in rheumatoid arthritis. Mol Diagn Ther.

[40] Moncrieffe H, Ursu S, Holzinger D, Patrick F, Kassoumeri L, Wade A (2013). A subgroup of juvenile idiopathic arthritis patients who respond well to methotrexate are identified by the serum biomarker MRP8/14 protein. Rheumatology.

[41] Berg JM, Tymoczko JL, Stryer L. Biochemistry. 7th ed. New York: WH Freeman and company; 2012. p. 802–07.

[42] McInnes IB, Thompson L, Giles JT, Bathon JM, Salmon JE, Beaulieu AD (2015). Effect of interleukin-6 receptor blockade on surrogates of vascular risk in rheumatoid arthritis: MEASURE, a randomised, placebo-controlled study. Ann Rheum Dis.

[43] Nambiar S, Truby H, Abbott RA, Davies PSW (2009). Validating the waist-height ratio and developing centiles for use amongst children and adolescents. Acta Pædiatrica.

[44] McCarthy HD, Ashwell M (2006). A study of central fatness using waist-to-height ratios in UK children and adolescents over two decades supports the simple message – “keep your waist circumference to less than half your height”. International J Obesity.

[45] Sidhu D, Naugler C (2012). Fasting time and lipid levels in a community-based population. Arch Intern Med.

[46] Andersen LB, Riddoch C, Kriemler S, Hills A (2011). Physical activity and cardiovascular risk factors in children. Br J Sports Med.

[47] Bailey DP, Boddy LM, Savory LA, Denton SJ, Kerr CJ (2012). Associations between cardiorespiratory fitness, physical activity and clustered cardiometabolic risk in children and adolescents: the HAPPY study. Eur J Pediatr.

